# Transcription Factor ZNF281: A Novel Player in Intestinal Inflammation and Fibrosis

**DOI:** 10.3389/fimmu.2018.02907

**Published:** 2018-12-11

**Authors:** Maria Pierdomenico, Franscesca Palone, Vincenzo Cesi, Roberta Vitali, Anna Barbara Mancuso, Salvatore Cucchiara, Salvatore Oliva, Marina Aloi, Laura Stronati

**Affiliations:** ^1^Pediatric Gastroenterology and Liver Unit, Department of Pediatrics, Sapienza University of Rome, Rome, Italy; ^2^Division of Health Protection Technologies, Territorial and Production Systems Sustainability Department, ENEA, Rome, Italy; ^3^Department of Cellular Biotechnology and Hematology, Sapienza University of Rome, Rome, Italy

**Keywords:** ZNF281, intestinal inflammation, fibrosis, DSS mice, inflammatory bowel disease, pediatric patients

## Abstract

**Background and aims:** Recent evidences reveal the occurrence of a close relationship among epithelial to mesenchymal transition (EMT), chronic inflammation and fibrosis. ZNF281 is an EMT-inducing transcription factor (EMT-TF) involved in the regulation of pluripotency, stemness, and cancer. The aim of this study was to investigate *in vitro, in vivo*, and *ex vivo* a possible role of ZNF281 in the onset and progression of intestinal inflammation. A conceivable contribution of the protein to the development of intestinal fibrosis was also explored.

**Methods:** Human colorectal adenocarcinoma cell line, HT29, and C57BL/6 mice were used for *in vitro* and *in vivo* studies. Mucosal biopsy specimens were taken during endoscopy from 29 pediatric patients with Crohn's disease (CD), 24 with ulcerative colitis (UC) and 16 controls. ZNF281 was knocked down by transfecting HT29 cells with 20 nM small interference RNA (siRNA) targeting ZNF281 (siZNF281).

**Results:** We show for the first time that ZNF281 is induced upon treatment with inflammatory agents in HT29 cells, in cultured uninflamed colonic samples from CD patients and in DSS-treated mice. ZNF281 expression correlates with the disease severity degree of CD and UC patients. Silencing of ZNF281 strongly reduces both inflammatory (IL-8, IL-1beta, IL-17, IL-23) and EMT/fibrotic (SNAIL, Slug, TIMP-1, vimentin, fibronectin, and α-SMA) gene expression; besides, it abolishes the increase of extracellular-collagen level as well as the morphological modifications induced by inflammation.

**Conclusions:** The identification of transcription factor ZNF281 as a novel player of intestinal inflammation and fibrosis allows a deeper comprehension of the pathogenetic mechanisms underlying inflammatory bowel disease (IBD) and provide a new target for their cure.

## Introduction

Inflammatory bowel disease (IBD) is a chronic inflammatory disorder of the gastrointestinal (GI) tract including two main phenotypes: Crohn's disease (CD) and ulcerative colitis (UC). Although many investigations have highlighted an abnormal interplay among genetic, immunity and gut microbiota as a key mechanism underlying IBD, a full understanding of IBD pathogenesis is still unclear (1). Intestinal fibrosis and subsequent stricture formation are frequent complications of IBD, particularly in CD. Current hypothesis suggests that the first step of intestinal fibrosis is a tissue damage caused by a chronic inflammatory state. Afterward, activated fibroblasts are recruited to the sites of inflammation to induce wound healing and, finally, fibrosis results due to excessive deposition of extracellular matrix (ECM) (2, 3). Recent studies propose that epithelial to mesenchymal transition (EMT), typically defined by the acquisition of a spindle cell morphology in combination with loss of E-cadherin, is involved in intestinal fibrosis following chronic inflammation (4–7).

Zinc-finger proteins (ZNFs) are one of the most abundant groups of proteins with a key role in the regulation of important cellular processes. Alterations in ZNFs are involved in the development of several human diseases and in the progression of cancer, including colorectal cancer (8, 9). Among ZNFs, ZNF281, also known as ZBP-99 or ZNP-99, is a 99 kDa Krüppel-type zinc-finger transcriptional regulator (10), expressed at high levels in placenta, kidney, brain, heart, liver, and lymphocytes, whereas most other tissues display detectable albeit low ZNF281 expression (11, 12).

Recently, ZNF281 has been characterized as an EMT-inducing transcription factor (EMT-TF), suggesting its involvement in the regulation of pluripotency, stemness, and cancer (11, 13–15). Moreover, it seems that ZNF281 is a direct SNAIL target required for SNAIL-induced EMT (16).

In the present study, we investigated *in vitro, in vivo* and *ex vivo* the role of ZNF281 in intestinal inflammation and in the regulation of pro-fibrotic genes. We showed that ZNF281 is induced by inflammatory agent exposure in intestinal cells, in murine colonic tissues and in cultured mucosal samples taken from uninflamed colonic areas of CD patients. Interestingly, ZNF281 silencing strongly reduces inflammatory as well as pro-fibrotic genes, including SNAIL. Furthermore, we demonstrated that ZNF281 and SNAIL are significantly increased, while E-cadherin decreased, in the inflamed colon of CD and UC patients, and ZNF281 expression levels correlate with the disease severity. Our data strongly suggest for the first time a role of ZNF281 in controlling intestinal inflammation and highlight its potential in eliciting an EMT-induced intestinal fibrosis.

## Materials and Methods

### Cell Culture

The human intestinal colorectal adenocarcinoma cell line, HT29, was used for cell culture experiments (ATCC, Rockville, MD, USA). Cells were maintained in 5% CO_2_ atmosphere at 37°C in Dulbecco's modified Eagle's medium with 2 mM L-glutamine supplemented with 10% fetal bovine serum. The pro-inflammatory cytomix, containing 250 ng/ml of IFNgamma (Sigma, St. Louis, MO) and 100 ng/ml of TNFalpha (Sigma), was added, for 24 h, for treatment of cell culture. All treatments were carried out in serum-free condition. After 24 h, proteins and RNA were extracted for western blot and real-time PCR analysis, respectively.

### Animals

C57BL/6 female mice, a model highly susceptible to DSS-induced colitis (8–9 weeks of age), were housed in collective cages at 22/21°C under a 12 h light/dark cycle and with food and water provided *ad libitum*. Acute colitis was induced through administration of dextran sodium sulfate (DSS, molecular mass, 36,000–50,000 Da, MP Biomedicals, Santa Ana, CA), dissolved in autoclaved drinking water, for 7 days. Mice, 6 for group, were randomly divided into two experimental groups: the control group received regular drinking water and the DSS-treated group received a solution with 3% (w/v) DSS. Mice were daily checked for behavior, body weight, stool blood, and consistency. The assessment of DSS-induced colitis and histological score were performed as previously described (17). The 7th day the animals were euthanized, colons were removed and frozen in liquid nitrogen for protein extraction.

### Patients

Twenty nine pediatric patients with CD (median age: 14.9 years; range: 6–18 years), 24 with UC (median age: 12.7 years; range: 6–18 years), and 16 controls (median age: 12.5 years; range: 6–18 years), referred to the Pediatric Gastroenterology and Liver Unit of the Department of Pediatrics of the Sapienza University of Rome-University Hospital Umberto I were included in this study. All patients had an established diagnosis of IBD in accordance with the ESPGHAN Porto Criteria (18) and needed an ileocolonoscopy due to a disease flare up despite therapy. The latter included azathioprine, mesalazine, antibiotics or oral corticosteroids at low doses. Patients undergoing biological therapy were excluded from the study. The intestinal inflammation was assessed at endoscopy by using the Simple Endoscopic Score for Crohn Disease (SES-CD) (19) and the endoscopic Mayo subscore (20) in CD and UC patients, respectively (Tables 1, 2). Children with incapacitating functional GI complaints, having normal endoscopy, served as controls (CTRL).

**Table 1:** Characteristics of the CD study population.

**Subject**	**Sex**	**Age (years)**	**SES-CD**	**Location**	**Therapy**
CD1	F	14	6	Inflamed colon	Azathioprine, mesalamine
CD2	M	9	15	Inflamed colon	None
CD3	M	12	12	Inflamed colon	None
CD4	M	14	30	Inflamed colon	None
CD5	M	6	30	Inflamed colon	Nutrition
CD6	M	16	7	Inflamed colon	Azathioprine, corticosteroids
CD7	M	13	21	Inflamed colon	Corticosteroids
CD8	M	18	25	Inflamed colon	Mesalamine
CD9	F	18	9	Inflamed+Uninflamed colon	Mesalamine
CD10	M	15	17	Inflamed colon	None
CD11	M	18	7	Inflamed colon	Mesalamine, antibiotics
CD12	M	18	12	Inflamed colon	Azathioprine
CD13	F	17	18	Inflamed colon	Mesalamine, nutrition
CD14	M	15	13	Inflamed+Uninflamed colon	None
CD15	M	14	13	Inflamed colon	Azathioprine
CD16	F	13	3	Inflamed colon	Corticosteroids
CD17	F	18	7	Inflamed colon	Azathioprine, corticosteroids
CD18	M	15	18	Inflamed colon	Corticosteroids
CD19	F	17	14	Inflamed colon	Antibiotics
CD20	M	17	20	Inflamed colon	Corticosteroids
CD21	F	17	10	Inflamed colon	Corticosteroids
CD22	M	10	18	Inflamed colon	Corticosteroids
CD23	M	15	8	Inflamed colon	Corticosteroids
CD24	F	17	0	Uninflamed colon	Mesalamine
CD25	M	14	2	Uninflamed colon	Azathioprine, mesalamine
CD26	M	14	0	Uninflamed colon	None
CD27	M	18	0	Uninflamed colon	None
CD28	M	18	5	Uninflamed colon	Azathioprine
CD29	F	14	0	Uninflamed colon	None

**Table 2:** Characteristics of the UC study population.

**Subject**	**Sex**	**Age (years)**	**Mayo score**	**Location**	**Therapy**
UC1	M	12	2	Inflamed colon	Azathioprine, sulphasalazine
UC2	F	18	2	Inflamed colon	Azathioprine
UC3	M	13	1	Inflamed colon	Azathioprine,sulphasalazine
UC4	F	10	2	Inflamed colon	Mesalamine
UC5	F	6	1	Inflamed+Uninflamed colon	None
UC6	F	11	1	Inflamed colon	None
UC7	F	10	1	Inflamed colon	Sulphasalazine
UC8	F	12	2	Inflamed colon	None
UC9	M	14	1	Inflamed+Uninflamed colon	Mesalamine, azathioprine
UC10	F	10	3	Inflamed colon	Mesalamine
UC11	M	9	2	Inflamed+Uninflamed colon	None
UC12	F	11	2	Inflamed colon	None
UC13	F	17	0	Inflamed colon	None
UC14	F	17	2	Inflamed+Uninflamed colon	None
UC15	F	15	2	Inflamed colon	None
UC16	F	16	2	Inflamed colon	Sulphasalazine
UC17	M	8	0	Inflamed colon	Azathioprine
UC18	M	7	2	Inflamed+Uninflamed colon	None
UC19	M	9	2	Inflamed colon	None
UC20	F	18	3	Inflamed colon	Mesalamine
UC21	F	17	1	Inflamed colon	Corticosteroids
UC22	M	18	1	Uninflamed colon	Mesalamine
UC23	F	13	1	Uninflamed colon	Mesalamine, corticosteroids
UC24	F	11	0	Uninflamed colon	Mesalamine

## Ethical Considerations

Experimental procedures *in vivo* were carried out in accordance with the recommendations of the Committee on the Ethics of Animal Experiments of the Italian National Agency for New Technology, Energy, and Sustainable Economic Development (ENEA - Permit Number: 1175/2016-PR).

All patients entered into the study after informed written consent from parents. The study was approved by the Ethical Committee of the Sapienza University of Rome.

### Biopsy Treatment

Mucosal biopsy specimens, taken from colonic districts, were immediately snap frozen in liquid nitrogen or cultured following the protocol described below.

### Organ Cultures

Mucosal biopsy specimens were taken during endoscopy from the colonic uninflamed areas of 3 CD patients. Two biopsy samples were taken from each patient and exposed to cytomix to induce inflammation. Untreated samples were used as controls. Biopsies were immersed in medium and cultured in 48-well plates containing 700 μL of Dulbecco's modified Eagle's medium supplemented with 10% fetal bovine serum, L-glutamine (2 mM), penicillin (100 U/ml), streptomycin (100 mg/ml), and gentamycin (100 mg/ml; Life Technologies- GibcoBRL, Milan, Italy). Inflammation was obtained by adding to the medium 250 ng/ml of IFNgamma and 100 ng/ml of TNFalpha for 24 h at 37°C and 5% CO2. Then, total proteins were extracted for western blot analysis.

### Protein Extraction

Mucosal biopsy specimens, cellular pellets, and mice colonic tissues were suspended in ice-cold lysis buffer (50 mM Tris (pH 7.4), 5 mM EDTA, 250 mM NaCl, 0.1% Triton X-100, 1 mM phenylmethylsulfonylfluoride, 5 μg/ml aprotinin, 5 μg/ml leupeptin, and 1 mM sodium orthovanadate), homogenized, and incubated in ice for 30 min. Samples were centrifuged at 14,000 rpm for 10 min and supernatants collected and analyzed by western blot.

### Immunoblot Analysis

30 μg of total proteins extracted from human biopsy and colonic tissue of mice, 15 μg of total protein extracted from HT29 cell line, 40 μl of culture supernatants were fractionated by sodium dodecyl sulfate-polyacrylamide gel electrophoresis. Proteins were transferred in polyvinylidene fluoride membrane (Amersham Pharmacia Biotech, Uppsala, Sweden) and blocked with TBS-T (Tris-buffered saline with 0.1% Tween-20) containing 5% non-fat dry milk. Anti-ZNF281 (1:1,000; Abcam, Cambridge, MA), anti-actin (1:1,000; Sigma), anti-SNAIL (1:1,000; Cell Signaling Technology, Danvers, MA), anti-E-cadherin (1:2,500, BD-Bioscience, Franklin Lakes, NJ), and anti-COL3A1 (1:000; Thermoscientific, Rockford, USA) were diluted in TBS-T containing 3% non-fat dry milk and incubated overnight at 4°C. Membranes were washed in TBS-T, incubated for 1 h with horseradish peroxidase-conjugated secondary antibody (Santa Cruz Biotechnology, CA, USA), washed in TBS-T and developed with Liteablot Extent Chemiluminescent substrate (Euroclone, MI, Italy). Densitometrical analyses of the blots were performed using the Software Image Quant Las 500 (GE Healthcare Life Science, Uppsala, Sweden).

### Immunohistochemistry

Paraffin-embedded intestinal mucosa sections (4 μm) of DSS-treated mice or controls were prepared following standard protocol. Briefly, sections were incubated in heat mediated antigen retrieval solution pH 6.0 (Abcam) for 10 min at 95°C and washed in water for 5 min. Peroxidases were then inhibited by incubations in 3% H_2_O_2_ for 10 min and sections were treated with 5% bovine serum albumin (Santa Cruz Biotech.) for 20 min, incubated with primary anti-ZNF antibody (Abcam) or anti-E-cadherin (BD Bioscience), diluted 1:100 or 1:250, respectively, in phosphate-buffered saline 5% BSA over night at 4°C in a moist chamber. After washing, sections were incubated with secondary anti-rabbit biotinylated antibody (Dako North America, Carpinteria, CA) or anti-mouse-HRP linked antibody (Santa Cruz Biotech.), for 30 min at room temperature. The DAB detection kit (Dako) was used to visualize the antigen. Finally, sections were stained with hematoxylin and eosin.

### Real Time PCR

Total RNA was isolated from HT29 cells using the RNeasy kit (QiaGen GmbH, Hilden, Germany), and 1 μg of total RNA was reverse transcribed by a IScriptTM cDNA Synthesis Kit (BioRad, Hercules, California, U.S.A). The RT-PCR amplifications were obtained by a BioRad CFX96 Touch™ Real-Time PCR Detection System using SsoAdvanced Universal SYBR Green super Mix (BioRad). The following primers were used: ZNF281 fwd primer 5′-GCCATCCTCTCCCCAAGTC-3′, rvs primer 5′-GAGCTTCGGAAAGCAGCACTA-3′; IL-8 fwd primer 5′-CTGGCCGTGGCTCTCTTG-3′, rvs primer 5′-CTTGGCAAAACTGCACCTTCA-3′; IL-17 fwd primer: 5′-GGGCCTGGCTTCTGTCTG-3′, rvs primer: 5′-AAGTTCGTTCTGCCCCATCA-3′; IL-1beta fwd primer: 5′-AGACATCACCAAGCTTTTTTGCT-3′, rvs primer: 5′-GCACGATGCACCTGTACGAT-3′; IL-23 fwd primer: 5′-TGCAAAGGATCCACCAGGGTCTGA-3′, rvs primer: 5′-TGAGTGCCATCCTTGAGCTGCTGC-3′; SLUG fwd primer: 5′-TGTGTGGACTACCGCTGCTC-3′, rvs primer: 5′- GAGAGGCCATTGGGTAGCTG-3′; SNAIL fwd primer: 5′-GACCACTATGCCGCGGTCTT-3′, rvs primer: 5′-TCGCTGTAGTTAGGCTTCCGATT-3′; TIMP-1 fwd 5′-CCATAACCCCGCAACTCACT-3′, rvs primer: 5′-AGGTAAGTGCCATGGTGAGC-3′; VIMENTIN fwd primer: 5′-CCTCCTACCGCAGGATGTT-3′, rvs primer: 5′-CTGCCCAGGCTGTAGGTG-3′; FIBRONECTIN fwd primer: 5′-AGACCATACCTGCCGAATGTAG-3′, rvs primer: 5′-GAGAGCTTCCTGTCCTGTAGAG-3′; α-SMA fwd primer: 5′-CCGACCGAATGCAGAAGGA-3′, rvs primer: 5′-ACAGAGTATTTGCGCTCCGAA-3′; GAPDH fwd primer 5′-GCACCGTCAAGGCTGAGAAC-3′, rvs primer 5′-GAGGGATCTCGCTCCTGG-3′. GAPDH expression level was used to normalize mRNA expression.

The quantity of mRNA relative to a reference gene was calculated by the 2^−Δ*CT*^ method.

### Small Interference RNA Transfection

1 × 10^5^ HT29 cells were seeded in a 6 multi-well plate to reach 50% confluence at time of transfection. After 24 h, cells were exposed to cytomix (IFNgamma 250 ng/ml + TNFalpha 10 ng/ml), then, transfected with 20 nM small interference RNA (siRNA) targeting ZNF281 (siZNF281) and negative control siRNA (siNC) (# 3898, Ambion, Carlsbad, USA) using the INTERFERin siRNA Transfection reagent (Polyplus transfection, Illkirch, France). Transfected cells were placed for 48 h at 37°C in 5% CO2 incubator. Cell pellets were collected for RNA and protein extraction and culture medium collected for ELISA. During procedures, representative images of cell morphology were acquired on a Leica DM IL LED inverted microscope with LED illumination Leica DMC2900 Digital camera and complimentary software Leica Application Suite (LAS) (magnification, 20X) (Leica Biosystems, Wetzlar, Germany).

### Quantification of Cytokines by Enzyme-Linked Immunosorbent Assay (ELISA)

The determination of IL-8, IL-1beta, IL-17, and IL-23 levels in cell culture medium was performed with a competitive ELISA kit (R&D, Minneapolis, USA), according to manufacturer's instructions. Changes in the O.D. absorbance were measured spectrophotometrically at a wavelength of 450 nm. The cytokine concentration was given as pg/ml.

### Statistics

All statistical analyses were performed with GraphPad InStat software (GraphPad Software, San Diego, CA, USA). The comparison between experimental groups was performed by unpaired non-parametric *t*-test (Mann–Whitney). Correlation between ZNF281 and endoscopic scores was assessed by the Spearman's rank correlation coefficient (r) according to the statistical distribution of analyzed population.

All experiments were repeated three times and are reproducible. Data were given as mean ± standard deviation (S.D). Differences were noted as significant ^*^p < 0.05, ^**^p < 0.01, ^***^p < 0.001.

## Results

### ZNF281 Gene/Protein Expression Increases in Inflamed Cells in *in vitro, in vivo*, and *ex vivo* Models

**Figure 1:**
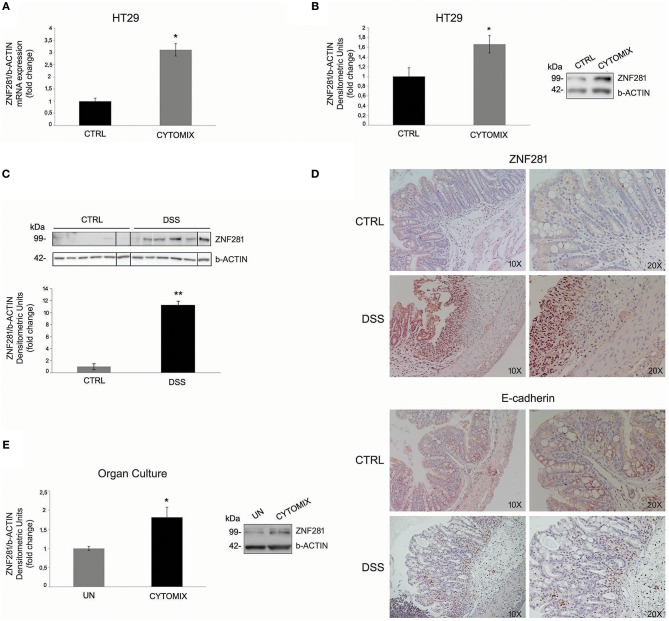
ZNF281 mRNA **(A)** and protein **(B)** expression levels are increased in HT29 cells exposed to cytomix as compared to control cells (CTRL); western blot **(C)** and immunohistochemistry **(D)** show that ZNF281 protein expression is significantly upregulated in the colonic mucosa of DSS-treated mice (*N* = 6) as compared to control (CTRL) mice (*N* = 6) (different gels were indicated); **(E)** ZNF281 protein expression is significantly increased in colonic mucosal tissues taken from healthy areas of 3 CD patients exposed to cytomix and cultured for 24 h, as compared to uninflamed (UN) colonic mucosal samples. Densitometric analysis is showed. Data represent the target gene expression normalized to the reference gene. Data are presented as mean ± S.D. of 3 *in vitro* and *ex vivo* independent experiments, and 6 animals for each group. CTRL, controls; Cytomix (TNFalpha + IFNgamma); DSS, dextran sodium sulfate; UN, uninflamed. Mann–Whitney *t*-test. ^*^P < 0.05; ^**^P < 0.01.

HT29 cells exposed to the cytomix (TNFalpha + IFNgamma) for 24 h showed a significant increase (*P* < 0.05) of ZNF281 mRNA and protein expression levels as compared to control cells (Figures 1A,B). To confirm this result *in vivo*, experimental colitis was induced in C57BL/6 mice by treatment with DSS 3% for 7 days. Untreated mice were used as controls. After 7 days, mice were sacrificed and the colon removed. The induction and severity degree of colitis was assessed by evaluating the following end-points: body weight loss, clinical score, histological score, large intestine length, and inflammatory cytokine expression (IL-6, IL-1beta) (Supplementary Figure 1). Western blotting analysis showed that ZNF281 protein expression was significantly (*P* < 0.01) upregulated in colonic mucosa of DSS-treated mice as compared to control mice (Figure 1C). Whole gels of *in vivo* experiments are provided as Supplementary Figure 2. Immunohistochemistry confirmed the strong ZNF281 up-regulation in epithelial cells and leukocyte infiltrate of inflamed tissues, and a parallel decrease of the EMT marker, E-cadherin (Figure 1D).

To further investigate this point, colonic mucosa samples taken from the uninflamed areas of 3 CD patients were exposed to cytomix and cultured for 24 h. Untreated samples were used as controls (UN). Results showed that ZNF281 protein expression was significantly increased (*P* < 0.05) in inflamed as compared to uninflamed colonic mucosal samples (Figure 1E).

### ZNF281 Is Increased in the Inflamed Colonic Tissues of CD and UC Pediatric Patients and Correlates With the Severity of the Disease

Mucosal colonic samples were taken during ileocolonoscopy from inflamed and uninflamed colonic mucosa of 29 CD (Table 1) and 25 UC (Table 2) patients as well as of 16 age-matched controls.

**Figure 2:**
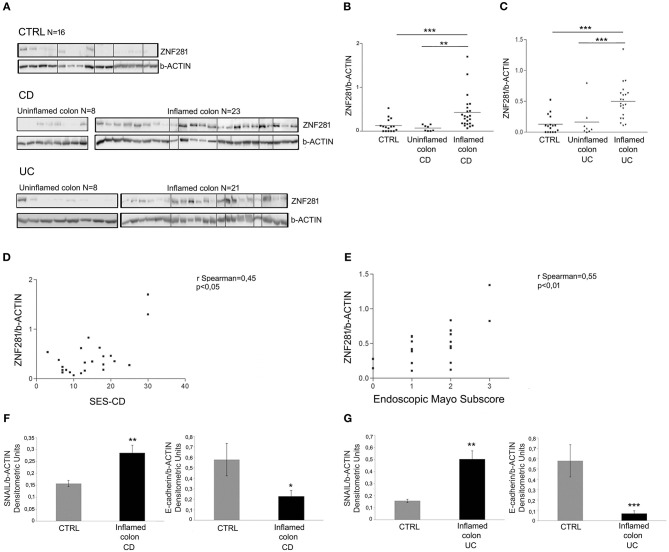
ZNF281 is increased in the inflamed colonic tissues of CD **(A,B)** and UC **(A,C)** pediatric patients as compared to uninflamed colonic samples and to controls (CTRL) (different gels were indicated); positive correlation between SES-CD, Mayo subscore and ZNF281 protein expression **(D,E)**; SNAIL protein expression is significantly increased and E-cadherin level decreased in inflamed colonic tissues of CD **(F)** and in UC **(G)** patients as compared to CTRL. Densitometric analysis is showed. Data represent the target gene expression normalized to the reference gene. Data are presented as mean ± S.D. of bioptic sample analyses from CD patients (*N* = 23), UC patients (*N* = 21)and age matched CTRL (*N* = 16). CTRL, controls; CD, Crohn Disease; UC, Ulcerative Colitis; SES-CD, Simple Endoscopic Score for Crohn's Disease. Mann–Whitney *t*-test; Spearman's rank correlation coefficient. *P < 0.05; **P < 0.01; ***P < 0.001.

We found a significant up-regulation of ZNF281 protein expression in the inflamed colon of CD (Figures 2A,B) and UC (Figures 2A,C) patients as compared to controls (*P* < 0.001) and to uninflamed colonic sample of the same patients (CD: *P* < 0.01, UC: *P* < 0.001). Whole gels of controls, CD and UC patients are provided as Supplementary Figures 3, 4.

Since the endoscopic score is currently considered a reliable measure of the intestinal inflammation degree, we related ZNF281 levels, expressed as densitometric values, to the SES-CD and to the endoscopic Mayo subscore for CD and UC patients, respectively. Results showed a positive correlation between SES-CD and ZNF281 protein expression (*r* = 0.45; *P* < 0.05) and between endoscopic Mayo subscore and ZNF281 protein expression (*r* = 0.55; *P* < 0.01), although, a full statistical significance was achieved only for the latter, probably due the low number of enrolled patients (Figures 2D,E).

Given the close relationship between ZNF281, SNAIL, and E-cadherin, all involved in EMT, we also investigated SNAIL and E-cadherin protein expression in mucosal colonic samples of inflamed CD, UC and in controls. Interestingly, we found that SNAIL protein level was significantly increased and E-cadherin decreased in CD (SNAIL: *P* < 0.01; E-cadherin *P* < 0.05) and UC (SNAIL: *P* < 0.01; E-cadherin *P* < 0.001) patients as compared to controls (Figures 2F,G).

### Silencing ZNF281 Significantly Decreases Inflammatory and Fibrotic Genes

**Figure 3:**
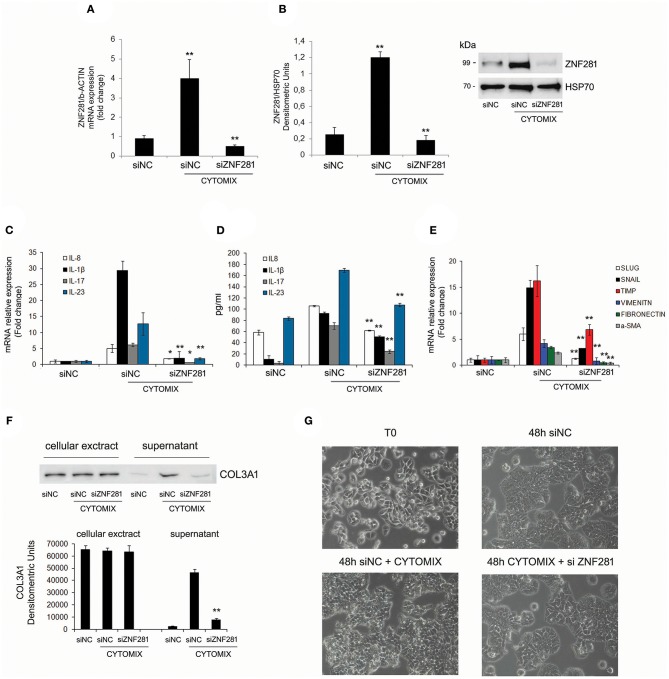
Exposure to cytomix (TNFalpha + IFNgamma) significantly increases mRNA **(A)** and protein **(B)** ZNF281 expression, that is strongly reduced after silencing; mRNA **(C)** and protein **(D)** expression of pro-inflammatory cytokines and mRNA expression **(E)** of EMT/pro-fibrotic genes are significantly decreased by ZNF281 knocking down. Western blot of cellular extracts and supernatants indicate that ZNF281 silencing in HT29 cells reduces the extracellular-collagen (COL3A1) deposition previously induced by 48 h of exposure to cytomix **(F)**. Optic microscopy images of changes in HT29 cell morphology after ZNF281 silencing **(G)**. Cytomix (TNFalpha + IFNgamma); siNC, negative control siRNA; siZNF281, ZNF281 targeted by small interference RNA. Mann–Whitney *t*-test. *P < 0.05; **P < 0.01; ***P < 0.001.

To further assess the role of ZNF281 in inflammation, a specific siRNA to knock-down ZNF281 expression was used. Thus, HT29 cells were exposed to the cytomix and transfected with a negative control siRNA (siNC) and siZNF281, for 48 h. Then, ZNF281 mRNA and protein expression was analyzed. Exposure to cytomix significantly up-regulated ZNF281 mRNA and protein levels (*P* < 0.01 and *P* < 0.001, respectively) that were strongly down-regulated by silencing (*P* < 0.01) (Figures 3A,B). Inflammation was assessed analyzing, by Real time PCR and ELISA, the following pro-inflammatory cytokines IL-8, IL-1beta, IL-17, and IL-23. Results showed that all cytokine mRNA and protein expression were significantly decreased after ZNF281 silencing (mRNA: IL-8, IL-17: *P* < 0.05, IL-1beta, IL-23: *P* < 0.01; protein: IL-8, IL-1beta, IL-17, and IL-23: *P* < 0.01) (Figures 3C,D). Furthermore, knock-down of ZNF281 significantly reduced mRNA expression of EMT and pro-fibrotic genes SLUG, SNAIL, TIMP-1, vimentin, fibronectin and α-SMA(*P* < 0.01) (Figure 3E), previously increased by inflammation. Finally, to highlight a functional effect of ZNF281 in EMT we analyzed the collagen deposition in the extracellular environment and the modification of cell morphology after ZNF281 silencing. We found that the exposure to cytomix induces after 48 h a significantly increase of extracellular-collagen gathering (*P* < 0.01) and a change in cell morphology that is abrogated by ZNF281 silencing (Figures 3F,G).

## Discussion

Intestinal fibrosis is a common complication of IBD: almost 30% of patients with CD and 5% of patients with UC undergo surgery due to intestinal strictures, with a recurrence risk around 50% (21, 22). Intestinal stenosis is detected in approximately 10-17% of children at the diagnosis, affecting up to 40% by 10 years after diagnosis (23).

Currently, it is rising the hypothesis that IBD-associated intestinal fibrosis might be the result of EMT. Transcription factor ZNF281, emerged as a critical regulator of EMT, has been recently thought to act at a nexus of inflammatory gene programs (24). Here, we provide the first evidence that ZNF281 is implicated in intestinal inflammation. Indeed, its gene/protein expression levels are strongly increased in inflamed HT29 cells, in inflamed colonic samples of CD patients cultured for 24 h and in mice with a DSS-induced colitis. To assess the active role of ZNF281 in inflammation, we knocked-down the gene in HT29 cells previously exposed to inflammatory agents and demonstrated that pro-inflammatory cytokines, such as IL-8, IL-17, IL-23, IL-1beta, are strongly down-regulated. We also found a significant increase of ZNF281 in inflamed colonic biopsies of CD and UC pediatric patients as compared to uninflamed samples of the same patients and to control subjects.

To our knowledge, a role of ZNF281 in inflammation had never been proven before. Our features could be relevant for more reasons. Firstly, the identification of a transcription factor that targeting effective inflammatory molecules is noteworthy as it might play a critical role in the pathogenesis of chronic inflammatory diseases. Secondly, ZNF281 could represent a novel therapeutic target for managing these complex disorders. Finally, since ZNF281 expression in the inflamed colonic mucosa of IBD patients positively correlates with the severity of the disease, as measured by the endoscopic indexes, it is tempting to suggest its use as a reliable marker of the inflammation degree.

More interestingly, we explored the potential of ZNF281 in promoting fibrosis, given the role of this protein in EMT and the link between EMT and fibrosis. Indeed, we found that, in addition to ZNF281, SNAIL is similarly increased and E-cadherin decreased in inflamed colonic tissues of CD and UC patients. These results are in accordance with previous observations supporting a relationship between ZNF281, SNAIL and E-cadherin; in particular, Hahn et al showed the occurrence of a coherent feed-forward loop between ZNF281 and SNAIL (16). Thus, since SNAIL is significantly up-regulated in inflamed HT29 cells and strongly reduced after ZNF281 silencing, we also suggest that ZNF281, known to be a SNAIL-target may in turn control SNAIL expression determining a sort of regulatory loop between the two proteins.

Moreover, we showed that the expression of EMT genes, SLUG, TIMP1, vimentin, fibronectin, and α-SMA, significantly increased during inflammation, is importantly decreased after ZNF281 silencing. This result is particularly striking because all these genes, in addition to their well-known role in EMT, have been recently implied in IBD fibrosis and complications. Accordingly, SLUG (SNAI2), a member of the SNAIL family of zinc finger transcriptional repressors, is a central regulator of EMT and acts by directly repressing E-cadherin (25). To date, studies have been mainly focused on the influence of the protein on tumor invasion and metastasis (26). However, more recently, several authors reported a strong increase of SLUG in inflamed and fibrotic tissues of UC and CD patients (4, 5) suggesting its role in EMT-induced fibrogenesis of IBD. Moreover, SNAIL and SLUG are thought to play a role in IBD complications, such as fistulae (27). Similarly, increased levels of TIMP-1, a member of tissue inhibitors of metalloproteinases (TIMP1-4) group, have been reported in fibrotic lesions of mice with chronic inflammation and CD patients (28–30) while TIMP-1 deficiency has been shown to weaken the development of fibrosis (31). In addition, the pro-fibrotic factors, vimentin, fibronectin, and α-SMA, have been recently found to be involved in fibrotic processes by cell transition to mesenchymal phenotype in IBD patients (32, 33).

As many studies focusing on the main phenotypic and functional alterations of fibrotic cells have stressed the relevance of improved collagen production, we analyzed the collagen deposition as well as the morphological change of inflamed intestinal cells before and after knocking-down ZNF281. We found that increased collagen amount in the extracellular matrix following inflammation is significantly reduced by ZNF281 silencing, likewise, intestinal cells assuming a more stretched shape revert to the initial morphology.

In conclusion, our study suggests a model whereby the transcription factor ZNF281 controls inflammatory genes, like IL-8, IL-17, IL-23, and IL-1beta, as well as EMT/pro-fibrotic factors, like SNAIL, SLUG, TIMP-1, fibronectin, vimentin, and α-SMA expression. During prolonged intestinal inflammation, increased ZNF281 promotes inflammation itself and potentially supports the EMT-induced fibrosis. Hence, if the pro-inflammatory role of ZNF281 will be confirmed *in vivo*, its inhibition could improve gut inflammation and fibrosis in subjects with chronic diseases, including IBD.

Future work will be aimed to deeply investigate the role of ZNF281 in the onset and progression of fibrosis.

## Author Contributions

LS and VC were involved in the conception and design of the study. SO and MA were involved in the enrollment of patients and acquisition of data. MP, FP and ABM made the experimental analysis. LS, MP and FP were involved in the interpretation of data. LS and RV were involved in drafting the article. SC and RV participated at the revision of the article.

### Conflict of Interest Statement

The authors declare that the research was conducted in the absence of any commercial or financial relationships that could be construed as a potential conflict of interest.

## Supplementary Material

Supplementary Figure 1 Assessment of DSS induced-colitis in mice. Analyzed parameters were analyzed: **(A)** weight loss; **(B)** colon length; **(C)** total clinical score (stool consistency, presence of blood in stool, and general appearance); **(D, E)** histology and histological score (10 × magnification in the image); **(F)** cytokine mRNA expression (IL-1beta, IL-6). Data are presented as mean ± S.D. of 6 animals for each group. UN, untreated animals; DSS, dextran sodium sulfate. Mann–Whitney *t*-test. ^*^*P* < 0.05; ^**^*P* < 0.01; ^***^*P* < 0.001.

Supplementary Figure 2 Whole gels of *in vivo* experiments.

Supplementary Figure 3 Whole gels of controls and CD patients.

Supplementary Figure 4 Whole gels of UC patients.
