# Supplementary figures and images for: Transcription Factor ZNF281: A Novel Player in Intestinal Inflammation and Fibrosis
Source: Front Immunol. 2018 Dec 11;9:2907. doi: 10.3389/fimmu.2018.02907 (PMC6297801; doi:10.3389/fimmu.2018.02907)

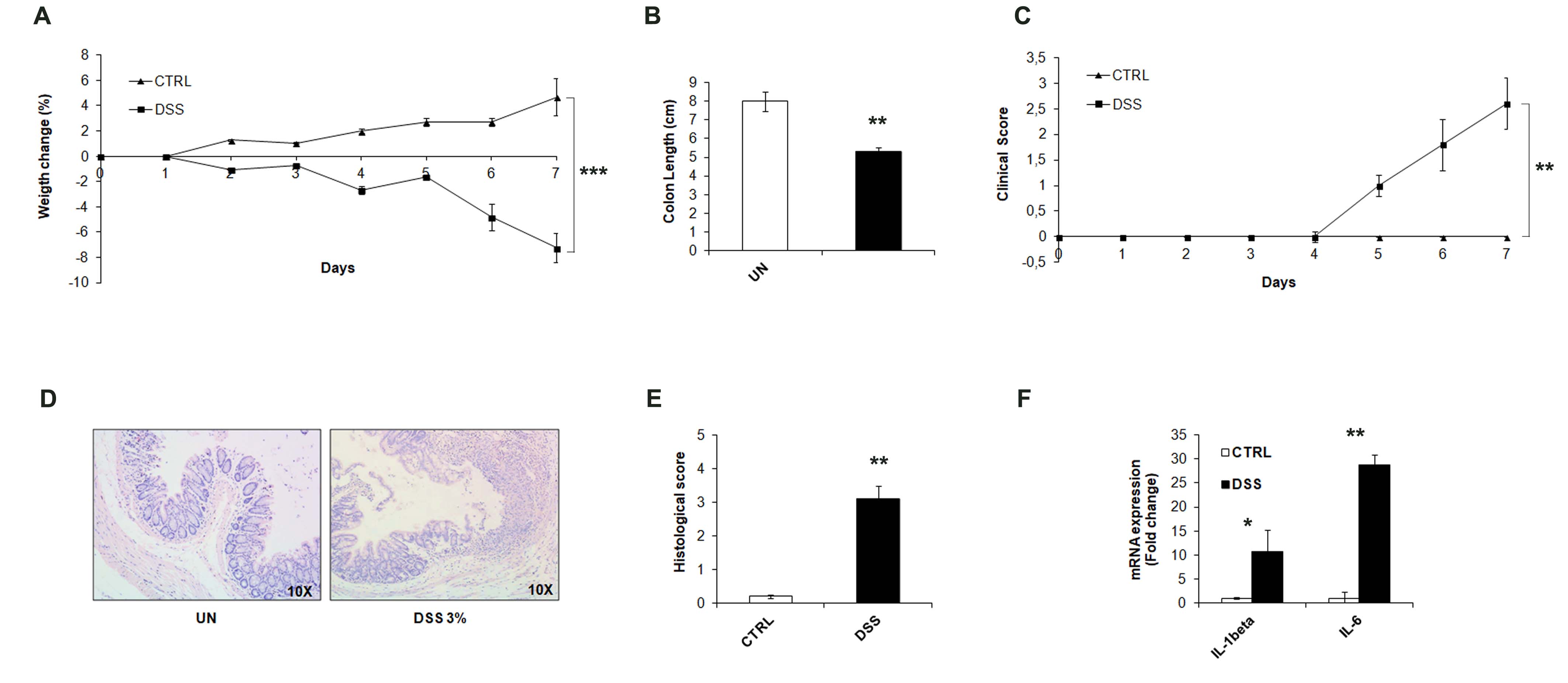

Supplement: Supplementary Figure 1 — Assessment of DSS induced-colitis in mice. Analyzed parameters were analyzed: (A) weight loss; (B) colon length; (C) total clinical score (stool consistency, presence of blood in stool, and general appearance); (D, E) histology and histological score (10 × magnification in the image); (F) cytokine mRNA expression (IL-1beta, IL-6). Data are presented as mean ± S.D. of 6 animals for each group. UN, untreated animals; DSS, dextran sodium sulfate. Mann–Whitney t-test. *P < 0.05; **P < 0.01; ***P < 0.001. [file Image_1.JPEG]

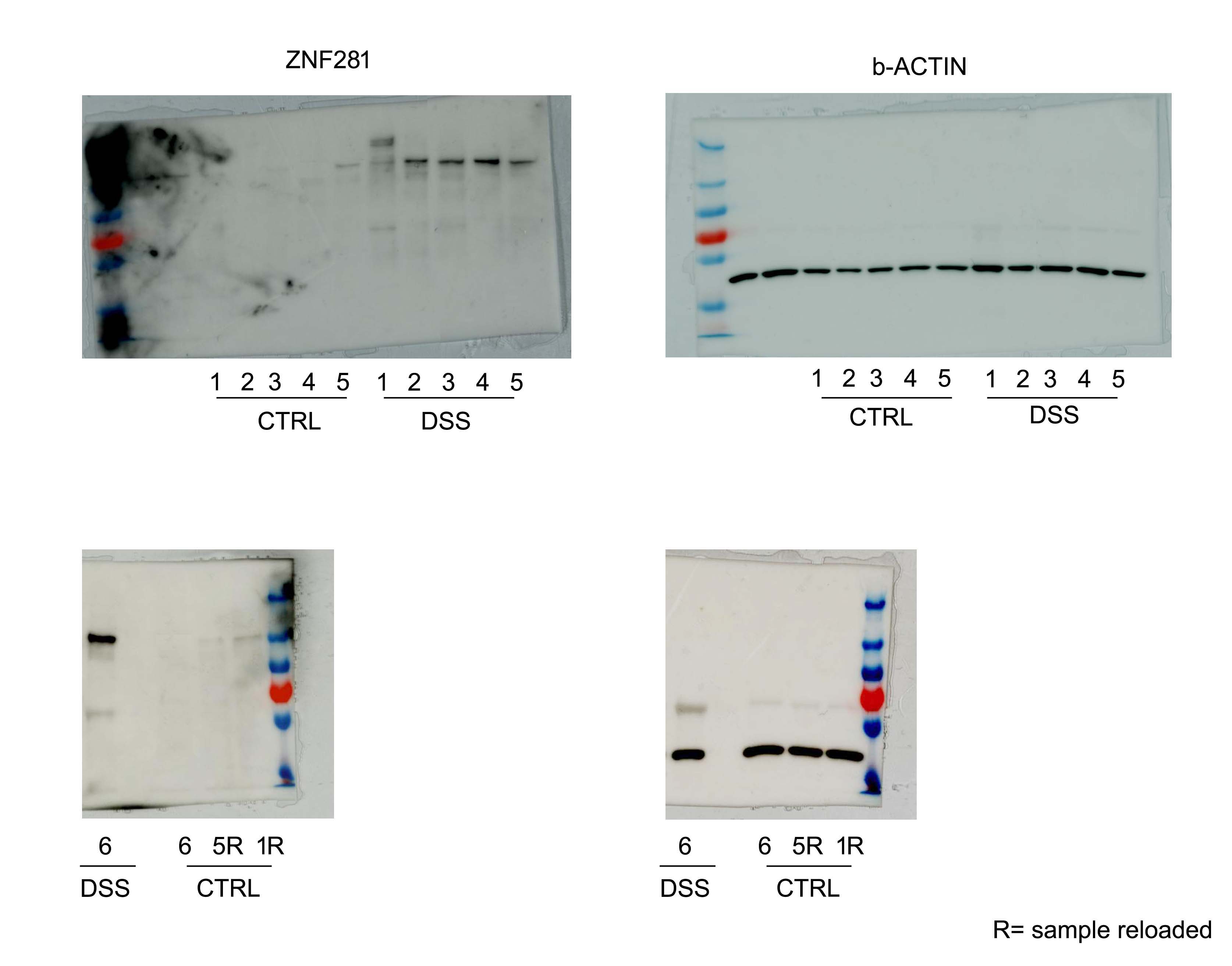

Supplement: Supplementary Figure 2 — Whole gels of in vivo experiments. [file Image_2.JPEG]

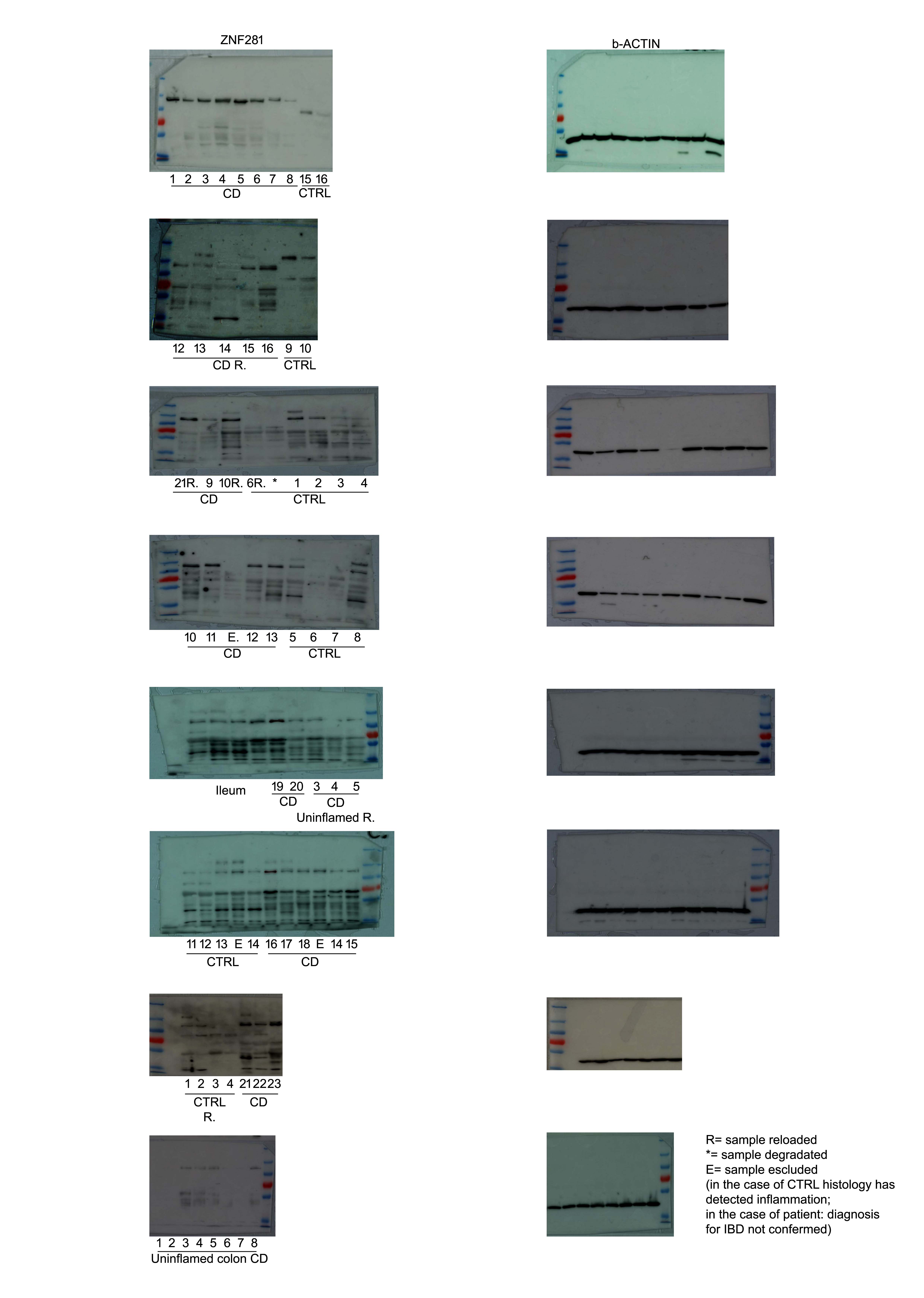

Supplement: Supplementary Figure 3 — Whole gels of controls and CD patients. [file Image_3.JPEG]

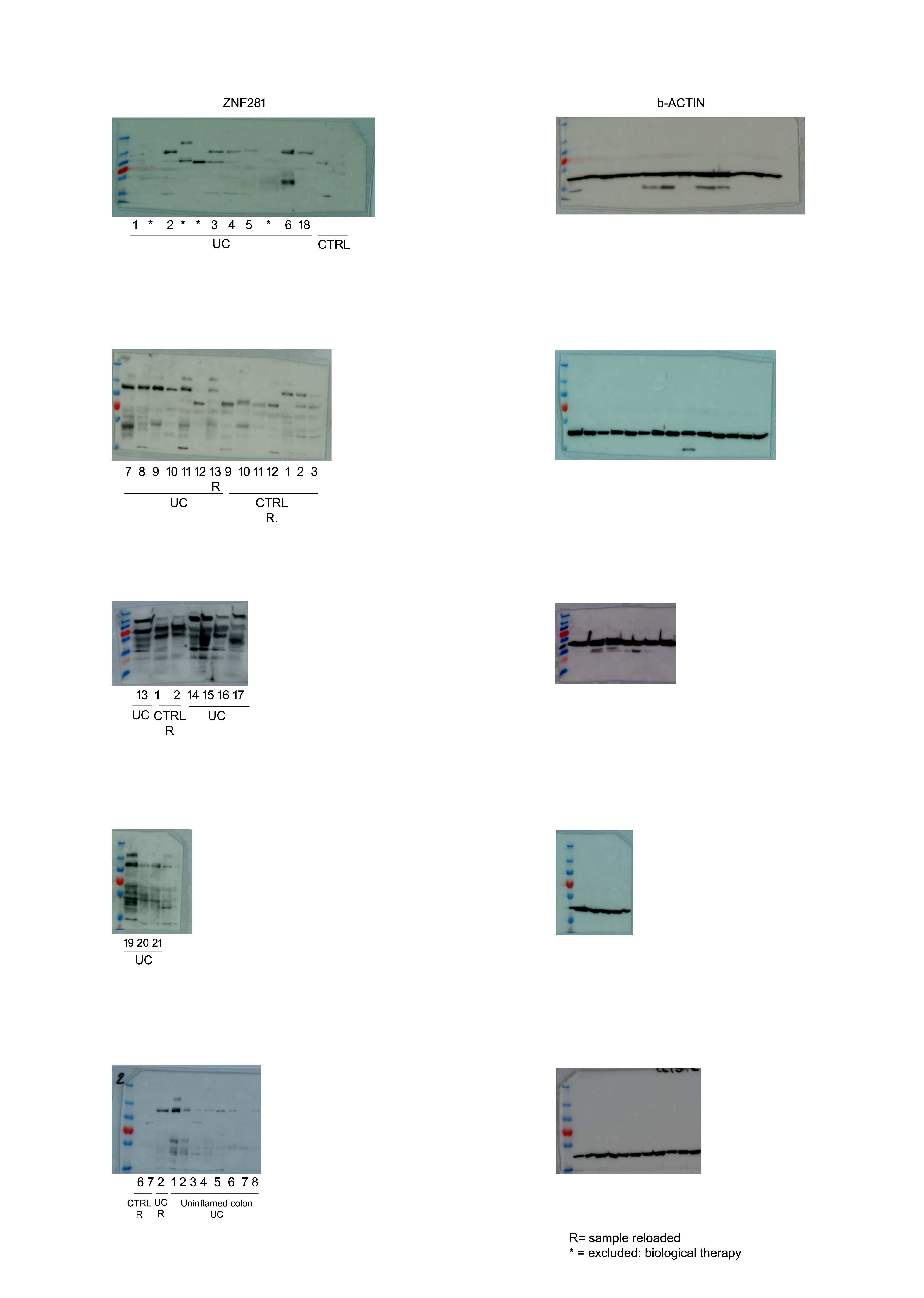

Supplement: Supplementary Figure 4 — Whole gels of UC patients. [file Image_4.JPEG]
